# Infrared laser moxibustion for cancer-related fatigue in breast cancer survivors: a randomized controlled trial

**DOI:** 10.1186/s13058-024-01838-1

**Published:** 2024-05-21

**Authors:** Huijuan Mao, Ming Jin, Lulu Xie, Ni Mao, Xubo Shen, Junchao Chen, Xuefen Chen, Jun J. Mao, Xueyong Shen

**Affiliations:** 1 School of Acupuncture-Moxibustion and Tuina, Shanghai University of Traditional Chinese Medicine, 1200 Cailun Road, Shanghai, 201203 China; 2 Shanghai University of Traditional Chinese Medicine, Yueyang Hospital of Integrated Traditional Chinese Medicine and Western Medicine, 110 Ganhe Road, Shanghai, 200437 China; 3 Institute of Disciplinary Science, Shanghai University of Traditional Chinese Medicine, 1200 Cailun Road, Shanghai, 201203 China; 4 School of Public Health, Shanghai University of Traditional Chinese Medicine, 1200 Cailun Road, Shanghai, 201203 China; 5 Bendheim Integrative Medicine Center, Memorial Sloan Kettering Cancer Center, 1429 First Avenue, New York, NY 10021 USA

**Keywords:** Acupuncture, Moxibustion, Infrared laser, Cancer-related fatigue, Breast cancer, Cancer survivor

## Abstract

**Background:**

Cancer-related fatigue (CRF) is a pervasive, persistent, and distressing symptom experienced by cancer patients, for which few treatments are available. We investigated the efficacy and safety of infrared laser moxibustion (ILM) for improving fatigue in breast cancer survivors.

**Methods:**

A three-arm, randomized, sham-controlled clinical trial (6-week intervention plus 12-week observational follow-up) was conducted at a tertiary hospital in Shanghai, China. The female breast cancer survivors with moderate to severe fatigue were randomized 2:2:1 to ILM (*n* = 56) sham ILM (*n* = 56), and Waitlist control (WLC)(*n* = 28) groups. Patients in the ILM and sham ILM (SILM) groups received real or sham ILM treatment, 2 sessions per week for 6 weeks, for a total of 12 sessions. The primary outcome was change in the Brief Fatigue Inventory (BFI) score from baseline to week 6 with follow-up until week 18 assessed in the intention-to-treat population.

**Results:**

Between June 2018 and July 2021, 273 patients were assessed for eligibility, and 140 patients were finally enrolled and included in the intention-to-treat analysis. Compared with WLC, ILM reduced the average BFI score by 0.9 points (95% CI, 0.3 to 1.6, *P* = .007) from baseline to week 6, with a difference between the groups of 1.1 points (95% CI, 0.4 to 1.8, *P* = .002) at week 18. Compared with SILM, ILM treatment resulted in a non-significant reduction in the BFI score (0.4; 95% CI, -0.2 to 0.9, *P* = .206) from baseline to week 6, while the between-group difference was significant at week 18 (0.7; 95% CI, 0.2 to 1.3, *P* = .014). No serious adverse events were reported.

**Conclusion:**

While ILM was found to be safe and to significantly reduce fatigue compared with WLC, its promising efficacy against the sham control needs to be verified in future adequately powered trials.

**Trial registration:**

Clinicaltrials.gov: NCT04144309. Registered 12 June 2018.

**Supplementary Information:**

The online version contains supplementary material available at 10.1186/s13058-024-01838-1.

## Background

Breast cancer remains the most prevalent form of cancer among women throughout the world, although therapeutic advances have led to improvements in the survival rates of patients [1]. Women with breast cancer can face high levels of persistent and disruptive cancer-related fatigue (CRF), which impacts an estimated 34–90% of these patients [2–5]. CRF can impair the ability of affected patients to function effectively, leading to reductions in their overall quality of life [6, 7]. Notably, CRF is also a significant predictor of recurrence-free survival among breast cancer survivors (BCS) [8]. Despite being a common finding in oncology patients, few treatment options for CRF are currently available due to the complexity of its etiology. While acupuncture is recommended as a treatment option for chemotherapy-associated nausea and vomiting by the Society for Integrative Oncology guidelines, the benefits of acupuncture as a treatment for CRF remain inconclusive [9]. The identification of effective acupuncture-based approaches to aiding patients affected by CRF with the adequate management of their fatigue symptoms is thus key to improving BCS quality of life.

Moxibustion is a form of acupuncture that, rather than relying on the use of needles to stimulate acupoints, employs the targeted burning of *Artemisia vulgaris*, a medicinal herb, above these acupoints in an effort to provide thermal stimulation and to thereby reduce symptom severity. Moxibustion can offer particular advantages to patients who may otherwise be hesitant to undergo acupuncture due to their concerns or anxieties related to needle puncture [10]. However, traditional moxibustion has several notable disadvantages including air pollution and the potential to burn the skin of the treated patients, limiting its broader appeal as a treatment for CRF. In contrast, infrared laser moxibustion (ILM) is a noninvasive procedure that provides the beneficial effects of traditional moxibustion without the associated pollution- or burn-related limitations. Our prior work has suggested that moxibustion can mediate its therapeutic benefits by delivering thermal radiation at particular wavelengths, with the peak wavelength generated by traditional moxibustion measuring ∼ 10 μm [11]. Accordingly, ILM employing a CO_2_ laser with a peak infrared radiation wavelength of 10.6 μm can mimic the effects of traditional moxibustion. In our preliminary research, ILM was found to be a safe and effective treatment for knee osteoarthritis [12], in addition to exhibiting promising efficacy when used to treat CRF [13].

Given our prior experience focused on this topic, we sought to conduct a sham-controlled randomized controlled trial (RCT) aimed at evaluating the safety and efficacy of ILM as a treatment for CRF among breast cancer survivors.

## Methods

### Trial design

This study was designed as a three-arm, parallel, RCT comparing ILM to sham ILM (SILM) or waitlist control (WLC) groups as a treatment for CRF among BCS. The trial design, recruitment/retention strategies, interventional approaches, and associated procedures have been published previously [14]. This trial received approval from the Institutional Review Board of Yueyang Hospital of Integrated Traditional Chinese Medicine and Western Medicine (IRB no. 2,018,029), and was performed in accordance with the Consolidated Standards of Reporting Trials (CONSORT) guidelines and the Standards for Reporting Interventions in Clinical Trials of Acupuncture (STRICTA).

### Patients

This trial enrolled female breast cancer (stage I-III) survivors 18 years of age or older at a minimum of 12 weeks following primary treatment (including surgery, radiotherapy, and/or chemotherapy). Enrolled patients were those complaining of persistent fatigue that was moderate-to-severe (≥ 4 on a numerical rating scale of average fatigue) despite having the ability to rest. Patients with fatigue that was potentially attributable to a treatable condition such as hypothyroidism or anemia were excluded. Participants were excluded if they had received acupuncture for any indication in the previous four weeks, had received an acupuncture or drug test within the past six months, were pregnant or lactating, were diagnosed with severe mental illness, or had a systemic infection or infectious diseases. All patients provided written informed consent before participating in the study.

### Randomization and masking

Potentially eligible patients were referred by oncology physicians who were contacted by research staff. In addition, trial-related information was posted on notice boards throughout Yueyang Hospital. Study clinicians met with interested patients to confirm that they met the criteria for eligibility. Those eligible patients that provided written informed consent were assigned to the ILM, SILM, or WLC groups at a 2:2:1 ratio, respectively. Randomization was achieved using a secure system with full allocation concealment, using permuted block randomization, with stratification by age and baseline level of fatigue. All statistical analyses were performed by statisticians blinded to the treatment assignments. The assessors and the statistician were blinded to treatment allocation throughout data collection and analysis. The study investigators, laser instrument operator, and participants were blinded to the treatment assignments between the real and sham laser moxibustion.

### Procedures and interventions

Participants in ILM and SILM groups were treated in a private room at the appointed time and were instructed not to talk to each other. All patients in three groups maintained their usual treatment and self-care during the study.

### Infrared laser moxibustion (ILM group)

Treatment of patients in the ILM and SILM groups was performed with SX10-C1 laser moxibustion devices (Shanghai Wonderful OptoElectrics Tech Co. Ltd., Shanghai, China). Four laser probes were simultaneously aligned with four selected acupoints (ST36 [bilateral], CV4, and CV6), with each of these points then being irradiated 2 cm from the surface of the skin for 20 min. For the ILM group, the output power level was 170 mW, the per-treatment energy density was 64.97 J/cm^2^, and the dose per treatment point was 203.91 J (eAppendix in Supplement Material). Treatment was performed twice per week for six weeks (12 total sessions) in all patients.

### Sham infrared laser moxibustion (SILM group)

The treatment protocol for patients in the SILM group was identical to that for patients in the ILM group, except that there was no laser emission when the moxibustion instrument was turned on. As the infrared laser used in the ILM group was colorless, it was not visible to the operator or the patient such that the procedure was double-blind.

### Waitlist control (WLC group)

No ILM or SILM treatment was provided to patients in the WLC group. These patients instead maintained standard treatment and self-care regimens, and did not initiate any additional treatments aimed at alleviating their CRF during the study period. After the completion of the study follow-up period, patients in the WLC group were provided with 10 real ILM treatments.

### Outcomes

The primary outcome of the study was a change in the mean patient score on the Chinese version of the Brief Fatigue Inventory (BFI-C) [15] over time. Specifically, changes in the BFI-C scores were assessed at the end of treatment (week 6) and the end of follow-up (week 18) relative to baseline. The BFI includes three fatigue severity-related questions that are rated from 0 (no fatigue) to 10 (fatigue as bad as you can imagine), as well as 6 questions related to the degree to which fatigue interferes with particular functions, rated from 0 (does not interfere) to 10 (interferes completely) [16]. The mean score for these 9 items was the primary outcome variable for this study.

The secondary outcomes included patients’ scores on the Pittsburgh Sleep Quality Index (PSQI) [17, 18], the Hospital Anxiety and Depression Scale (HADS) [19], 10-item Perceived Stress Scale (PSS-10) [20], the Brief Pain Inventory (BPI) [21], and the Functional Assessment of Cancer Therapy-Breast (FACT-B) [22, 23]. These assessments were completed at baseline (week 0) and at weeks 3, 6, 12, and 18.

Moreover, the Acupuncture Expectancy Scale (AES) [24, 25] was also used to gauge participant response expectancy regarding laser moxibustion for patients in the ILM and SILM groups at baseline (week 0), week 3, and week 6 (end of treatment). A standardized adverse-event case report form was completed by research staff at each ILM/SILM treatment visit during the interventional period, and adverse events were also recorded during subsequent patient follow-up.

### Statistical analysis

In our prior study, we found that ILM treatment yielded a significantly greater 0.60 standard deviation (SD) reduction in BFI-C scores at the end of a 4-week interventional period relative to baseline when compared to SILM treatment. With a two-sided alpha of 0.05, 45 subjects would thus be required in each of the ILM and SILM groups to detect a 0.60 SD reduction in BFI-C scores between these two groups at an 80% power level. By enrolling 45 subjects in each of the ILM and SILM groups and 22 subjects in the WLC group, it would be possible to detect a 0.71 SD change in BFI-C scores between the ILM and SILM or WLC groups at an 80% power level with a two-sided alpha of 0.05. Assuming that 20% of patients would be lost to follow-up, the study thus planned to enroll 140 patients that would be assigned to the ILM, SILM, and WLC groups at a 2:2:1 ratio. As the study used repeated measures and mixed-effects models to analyze the resultant data, providing higher statistical power than t-tests, this was a relatively conservative approach to study power calculation.

The study was conducted based on an intention-to-treat (ITT) principle. Baseline characteristics were compared among groups with one-way ANOVAs, Kruskal-Wallis and chi-square tests, or Fisher’s exact test. Changes in the BFI and their interactions with group assignment and time were evaluated using a linear mixed-effects model. Linear mixed-effects models were also used to assess the secondary PSQI, HADS, PSS-10, BPI, and FACT-B outcome measures. Linear mixed-effects models enable valid inferences based on the assumption that any missing follow-up data are missing at random. Missing values were thus not replaced for these analyses. Results were corrected for multiple comparisons via the Bonferroni correction, and a two-sided *P* < .05 was the threshold for significance in all analyses.

## Results

### Participant characteristics

**Fig. 1:**
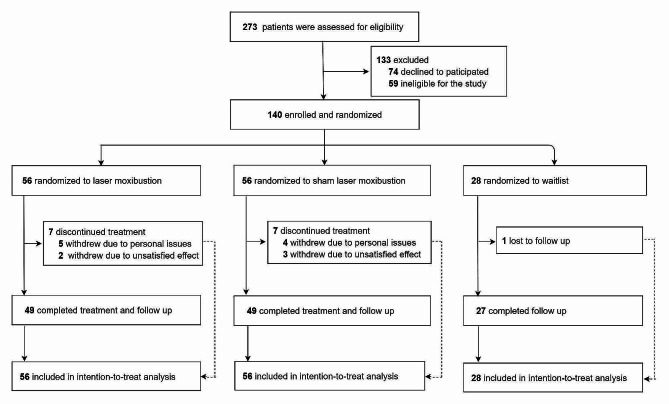
Participant flow

A flow diagram for the selection of the study participants is provided in Fig. 1. Between June 2018 and July 2021, 273 BCS were screened, of whom 133 were ineligible or declined to participate in the study (Fig. 1). Of the remaining 140 patients who were enrolled, 56, 56, and 28 were randomly assigned to the ILM, SILM, and WLC groups, respectively. Of the patients in the ILM and SILM groups, 49 (87.5%) in each group completed all treatments and follow-up. Overall, 15 patients (10.7%) withdrew from data collection at week 6.

The demographic and clinical characteristics of the patients in the 3 groups were similar at baseline. The mean (SD) age of the patients was 59.4 (8.3) years. The mean (SD) baseline BFI scale score was 3.8 (1.5) points (Table 1).

**Table 1:** Baseline characteristics of patients

Characteristics	ILM (*n* = 56)	SILM (*n* = 56)	WLC (*n* = 28)
Age, mean (SD), y	60.5 (6.8)	58.2 (8.7)	59.5 (9.9)
Education, N (%)			
High school or less	40 (71.4%)	40 (71.4%)	17 (60.7%)
College or more	16 (28.6%)	16 (28.6%)	11 (39.3%)
Employment status, N (%)			
Employed	1 (1.8%)	5 (8.9%)	3 (10.7%)
Retired	55 (98.2%)	51 (91.1%)	25 (89.4%)
Marital status, N (%)			
Married	48 (85.7%)	53 (94.6%)	21 (75.0%)
Single/Divorced/Widowed	8 (14.3%)	3 (5.4%)	7 (25.0%)
Body Mass Index, mean (SD), Kg/m^2^	24.0 (3.3)	22.9 (2.7)	23.4 (3.4)
Months since cancer diagnosis, median (Q_1_,Q_3_)	28.0 (14.5, 60.0)	28.5 (18.0, 58.3)	40.0 (20.5, 68.5)
Cancer Stage, N (%)			
I	12 (21.4%)	8 (14.3%)	10 (35.71%)
II	29 (51.8%)	31 (55.4%)	13 (46.4%)
III	15 (26.8%)	17 (30.4%)	5 (17.9%)
Previous treatments, N (%)			
Surgery	56 (100.0%)	56 (100.0%)	28 (100.0%)
Chemotherapy	44 (78.6%)	49 (87.5%)	21 (75.0%)
Radiotherapy	20 (35.7%)	27 (48.2%)	10 (35.7%)
Hormnal therapy	4 (7.1%)	5 (8.9%)	3 (10.7%)
Present hormonal treatments, N (%)	34 (60.7%)	39 (69.6%)	17 (60.7%)
Number of Comorbidities, N (%)			
0	14 (25.0%)	13 (23.2%)	8 (28.6%)
1	34 (60.7%)	40 (71.4%)	16 (57.1%)
2 or more	8 (14.3%)	3 (5.4%)	4 (14.3%)
Baseline Characteristics of Patients, mean (SD) or median (Q_1_,Q_3_)			
BFI	4.1 (1.6)	3.6 (1.4)	3.9 (1.3)
PSQI	9.8 (4.6)	9.5 (3.9)	9.0 (3.6)
FACT-B	103.3 (15.3)	104.1 (14.5)	104.2 (13.0)
HADS-A	3.5 (0.3, 6.0)	3.0 (1.0, 6.0)	3.5 (1.0, 7.0)
HADS-D	3.0 (1.0, 5.0)	2.0 (1.0, 5.0)	3.0 (1.0, 8.8)
PSS-10	11.0 (7.0, 15.0)	11.0 (6.0, 16.8)	12.0 (8.0, 16.8)
BPI	1.1 (0.0, 2.4)	1.3 (0.4, 3.4)	1.6 (0.2, 2.7)
AES	16.2 (3.4)	16.2 (3.2)	/

Abbreviations: ILM: Infrared Laser Moxibustion; SILM: Sham Infrared Laser Moxibustion; WLC: Waitlist Control; BFI: Brief Fatigue Inventory; PSQI: Pittsburgh Sleep Quality Index; ACT-B: Functional Assessment of Cancer Therapy-Breast; HADS: Hospital Anxiety and Depression Scale; PSS-10: 10-item Perceived Stress Scale; BPI: Brief Pain Inventory; AES: Acupuncture expectancy Scale

### Primary outcome

**Fig. 2:**
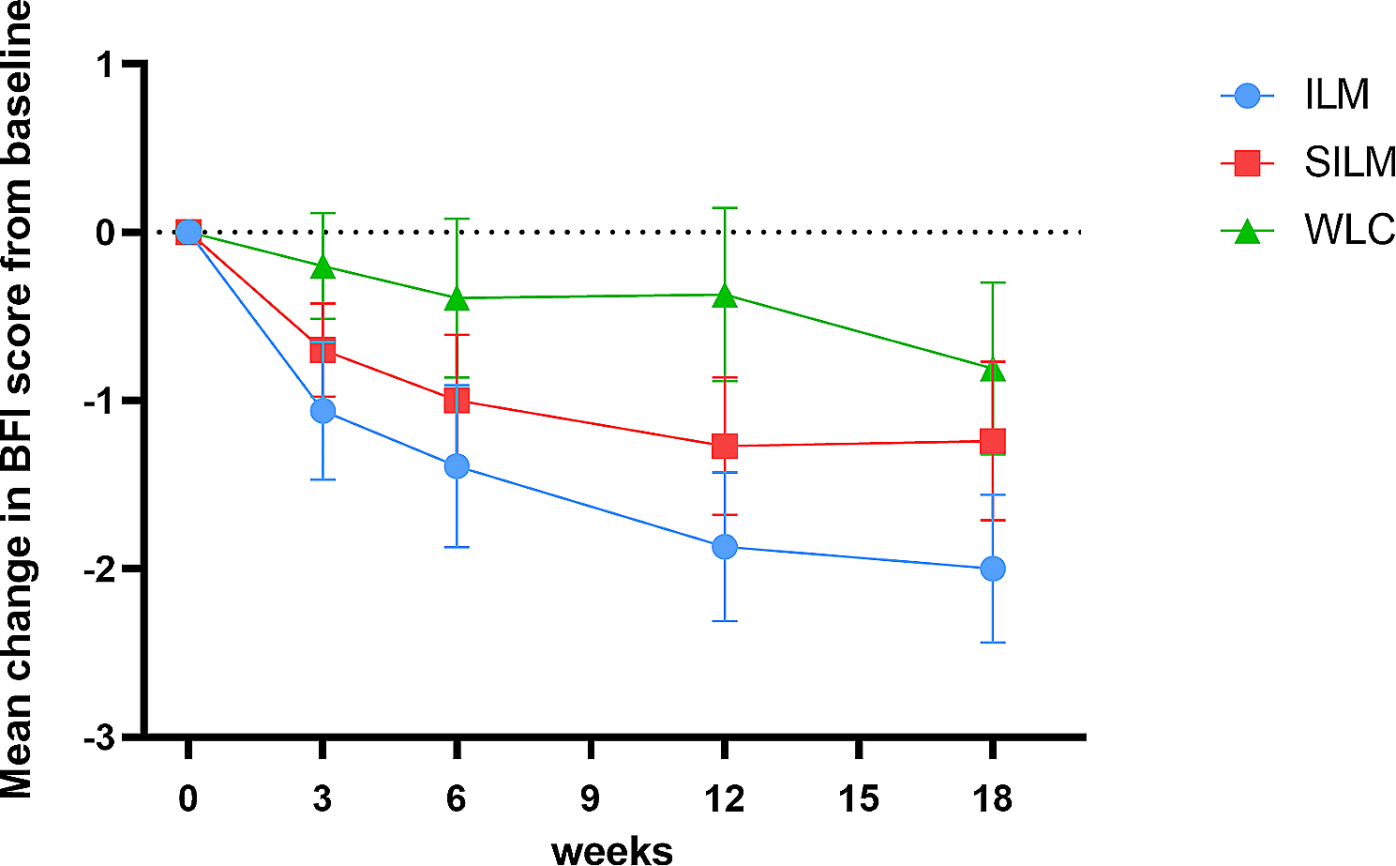
Mean change in BFI score from baseline over time. Error bar represents 95% confidence interval (CI)

Compared with WLC, ILM reduced fatigue, measured by the average BFI score, by 0.9 points (95% CI, 0.3 to 1.6, *P* = .007) from baseline to week 6, and the effect was maintained at week 18 (1.1, 95% CI, 0.4 to 1.8, *P* = .002) (Table 2). Therefore, compared with SILM, ILM resulted in a non-significant reduction in the BFI score (0.4; 95% CI, -0.2 to 0.9, *P* = .206) from baseline to week 6, although a significant reduction in the BFI score was found at week 18 (0.7, 95% CI, 0.2 to 1.3, *P* = .014) (Table 2; Fig. 2).

**Table 2:** Primary outcome across all study groups

Variable	Mean change in BFI score from baseline (95% CI)				ILM vs. SILM groups		ILM vs. WLC groups	
	ILM (*n* = 56)	SILM (*n* = 56)	WLC (*n* = 28)		Difference (95% CI)	^a^*P* value	Difference (95%CI)	^a^*P* value
week 3	-1.06 (-1.47,-0.66)	-0.70 (-0.96,-0.43)	-0.20 (-0.50,0.10)		-0.35 (-0.78,0.09)	0.1189	-0.80 (-1.32,-0.28)	0.0027
week 6	-1.39 (-1.87,-0.92)	-1.00 (-1.39,-0.62)	-0.39 (-0.84,0.06)		-0.36 (-0.92,0.20)	0.2060	-0.91 (-1.58,-0.25)	0.0072
week 12	-1.87 (-2.29,-1.44)	-1.27 (-1.66,-0.87)	-0.37 (-0.87,0.12)		-0.57 (-1.12,-0.03)	0.0392	-1.41 (-2.06,-0.76)	< 0.0001
week 18	-2.00 (-2.44,-1.57)	-1.24 (-1.70,-0.78)	-0.81 (-1.30,-0.31)		-0.74 (-1.32,-0.15)	0.0143	-1.11 (-1.81,-0.41)	0.0020

Abbreviations: ILM: Infrared Laser Moxibustion; SILM: Sham Infrared Laser Moxibustion; WLC: Waitlist Control; BFI: Brief Fatigue Inventory

^a^*P* values were calculated using a mixed-effects model

### Secondary outcomes

Compared with WLC, ILM resulted in a significant improvement in sleep quality, measured by the PSQI score, at week 6 (2.5, 95% CI, 1.1 to 3.9; *P* < .001), and the effect was still apparent at week 18 (Table 3). Therefore, compared with SILM, ILM led to a non-significant reduction in the PSQI score (1.0, 95% CI, -0.2 to 2.2, *P* = .111) from baseline to week 6, while a significant reduction in the PSQI score was found at week 18 (Table 3). Whether compared to WLC or SILM, ILM resulted in non-significant improvements in the FACT-B score at week 6 (Table 3). For further details regarding primary and secondary study outcomes, see eTables 1, 2 and 3 in the Supplementary data.

**Table 3:** Secondary outcomes across all study groups

Outcome	Mean change from baseline (95% CI)				ILM vs. SILM groups		ILM vs. WLC groups	
	ILM (*n* = 56)	SILM (*n* = 56)	WLC (*n* = 28)		Difference (95%CI)	^a^*P* value	Difference (95%CI)	^a^*P* value
**PSQI**								
week 3	-1.24 (-2.09,-0.40)	-0.55 (-1.38,0.28)	0.44 (-0.50,1.38)		-0.62 (-1.72,0.49)	0.2740	-1.60 (-2.92,-0.28)	0.0176
week 6	-2.06 (-2.93,-1.19)	-0.98 (-1.80,0.16)	0.59 (-0.75,1.94)		-0.97 (-2.16,0.23)	0.1111	-2.51 (-3.92,-1.10)	0.0006
week 12	-2.12 (-3.15,-1.09)	-1.08 (-2.01,-0.15)	-0.19 (-1.60,1.23)		-0.91 (-2.27,0.44)	0.1857	-1.70 (-3.31,-0.10)	0.0373
week 18	-3.00 (-4.06,-1.94)	-1.00 (-2.00,-0.00)	-0.70 (-1.78,0.38)		-1.85 (-3.18,-0.51)	0.0070	-2.11 (-3.70,-0.52)	0.0097
**FACT-B**								
week 3	3.88 (0.25,7.50)	2.67 (0.87,4.52)	0.37 (-1.49,1.56)		1.03 (-2.51,4.57)	0.5661	3.64 (-0.56,7.84)	0.0885
week 6	6.73 (2.52,10.95)	2.80 (-0.12,5.60)	1.22 (-1.12,3.56)		3.72 (-0.70,8.14)	0.0987	5.12 (-0.02,10.45)	0.0510
week 12	7.27 (3.16,11.37)	5.24 (3.19,7.29)	2.22 (-0.99,5.44)		1.80 (-2.34,5.94)	0.3919	4.82 (-0.09,9.73)	0.0545
week 18	7.86 (3.94,11.77)	4.39 (1.24,7.54)	2.44 (-0.39,5.28)		3.26 (-1.21,7.72)	0.1518	5.12 (-0.19,10.43)	0.0585

Abbreviations: ILM: Infrared Laser Moxibustion; SILM: Sham Infrared Laser Moxibustion; WLC: Waitlist Control; PSQI: Pittsburgh Sleep Quality Index; FACT-B: Functional Assessment of Cancer Therapy-Breast.

^a^*P* values were calculated using a mixed-effects model

### Adverse events

No serious adverse events were reported in any groups. Four of the patients in the ILM group exhibited localized erythema ∼ 5 mm in diameter below the laser probe following treatment, but this was resolved within 3 days without any specific treatment in all cases. None of the patients in the SILM or WLC groups reported any adverse events. No patients withdrew from the study due to adverse events.

## Discussion

Persistent fatigue is frequently reported as a distressing symptom among BCS. This RCT found that ILM treatment resulted in statistically significant and clinically relevant improvements in both fatigue and insomnia among BCS, compared to standard care. While ILM did not outperform sham treatment at the week-6 endpoint, it did significantly improve fatigue and insomnia relative to SILM at the week-18 follow-up time point. This suggests that ILM may mediate some level of delayed effect that persists beyond the placebo effect. These results offer promise as they underscore a potentially viable approach to managing and treating fatigue and associated symptoms among cancer survivors.

These results align well with a growing body of literature demonstrating the ability of acupuncture and moxibustion to help alleviate the fatigue [26, 27] and insomnia [28, 29] experienced by patients diagnosed with or recovering from cancer. Strikingly, ILM failed to significantly improve fatigue as compared to sham treatment at the end of the treatment interval despite exhibiting significant benefits at later follow-up time points, consistent with the results of another recent moxibustion trial [27]. This suggests that ILM can exhibit prolonged benefits as a treatment for CRF, although the underlying mechanisms remain to be fully clarified. In one pilot trial, moxibustion was found to be superior to acupuncture as a long-term treatment for fatigue, leading researchers to speculate that the beneficial effects of moxibustion may be related to modulation of the vagus nerve [30]. We also cannot ignore that the removing of blinding after week 6 could have influenced this delayed effect. Patients in the ILM group in this study exhibited a > 30% decline in the mean BFI scores from week 6 relative to baseline (34% at week 6, 46% at week 12, and 49% at week 18, respectively), with a reduction of this magnitude considered clinically meaningful [31].

CRF is a common symptom experienced by cancer patients and individuals undergoing treatment for cancer, with high rates of severe fatigue and insomnia irrespective of the cancer type and/or stage throughout all treatment phases [32]. According to the established cutoffs of these questionnaires, the participants did not experience any significant anxiety (cutoff score: >7),depression (cutoff score: >7), or mild pain (cutoff score: >3). A total PSS-10 score ranges between 0 and 40, with higher scores representing higher levels of stress. Although the PSS-10 has not published any specific score cut-offs, the participants in the present study appeared to have low levels of stress. The patients exhibited moderate fatigue and clinically significant insomnia, defined by a global score cut-off of 8 used to detect sleep disturbances among cancer patients [18]. ILM led to significant and meaningful improvements in sleep quality (mean PSQI score < 8 at weeks 12 and 18). As with the fatigue results discussed above, the observed improvements in sleep quality over the sham treatment were more pronounced at week 18, suggesting that ILM may exert delayed efficacy that warrants further investigation into its mechanism. These data indicate that ILM is an effective approach to managing the comorbid fatigue and insomnia that often impact BCS.

Low-level laser therapy (LLLT) induces a photobiomodulation effect which is used for the treatment of various diseases and conditions [33]. The light used in LLLT includes a CO_2_ laser at a wavelength of 10.6 μm, which was used in our study [34]. Infrared laser therapy is a non-invasive method that promotes pain relief and reduces inflammation, together with enhancing healing and tissue repair processes [35]. The etiology and pathogenesis of CRF is complex, with inflammation known to play an important role [36, 37]. It has been suggested that the mechanism underlying the effects of moxibustion on fatigue may involve the regulation of inflammatory cytokines [38]. We suggest that the potential mechanism of ILM on CRF may be related to anti-inflammatory processes. Infrared laser moxibustion mimics the effects and avoids the shortcomings of traditional moxibustion, and represents a promising and innovative non-pharmacological therapy for managing CRF. Therefore, further studies are required to explore the underlying therapeutic mechanisms of ILM.

### Limitations

This RCT is subject to certain limitations. Although our original study was powered to detect a difference in fatigue and sleep disorder between ILM and WLC, it was not powered to detect a statistically significant difference between ILM and the sham control. The promising delayed effect between ILM and the sham control was not observed at the primary end point, which is hypothesis-generating rather than confirming. The study was conducted on breast cancer survivors, and the efficacy of this intervention in patients receiving active treatment or survivors of other types of cancer is unknown. Additionally, we used validated instruments to measure fatigue as well as sleep quality but did not include objective measurement or biomarkers. Lastly, this study was conducted in China and thus further research is needed to determine its generalizability to other populations.

## Conclusion

In this randomized clinical trial, infrared laser moxibustion effectively improved fatigue and insomnia in female breast cancer survivors compared with usual care. However, the promising effect against the sham control requires further evaluation in an adequately powered trial.

### Electronic supplementary material

Below is the link to the electronic supplementary material.

Supplementary Material 1

Supplementary Material 2

## Data Availability

The datasets used during the current study are available from the corresponding author on reasonable request.

## Abbreviations

AES: Acupuncture expectancy Scale
BCS: Breast cancer survivors
BFI: Brief Fatigue Inventory
CRF: Cancer-related fatigue
FACT-B: Functional Assessment of Cancer Therapy-Breast
ILM: Infrared laser moxibustion
LLLT: Low-level laser therapy
PSQI: Pittsburgh Sleep Quality Index
SILM: Sham infrared laser moxibustion
SD: Standard deviation
WLC: Waitlist control

## References

[1] Bray F, Ferlay J, Soerjomataram I (2018). Global cancer statistics 2018: GLOBOCAN estimates of incidence and mortality worldwide for 36 cancers in 185 countries. CA Cancer J Clin.

[2] Palesh O, Scheiber C, Kesler S (2018). Management of side effects during and post-treatment in breast cancer survivors. Breast J.

[3] Mao H, Bao T, Shen X (2018). Prevalence and risk factors for fatigue among breast cancer survivors on aromatase inhibitors. Eur J Cancer.

[4] Fabi A, Falcicchio C, Giannarelli D (2017). The course of cancer related fatigue up to ten years in early breast cancer patients: what impact in clinical practice?. Breast.

[5] Bower JE, Ganz PA, Desmond KA (2006). Fatigue in long-term breast carcinoma survivors: a longitudinal investigation. Cancer.

[6] Curt GA (2000). Impact of fatigue on quality of life in oncology patients. Semin Hematol.

[7] Montazeri A (2008). Health-related quality of life in breast cancer patients: a bibliographic review of the literature from 1974 to 2007. J Exp Clin Cancer Res.

[8] Groenvold M, Petersen MA, Idler E (2007). Psychological distress and fatigue predicted recurrence and survival in primary breast cancer patients. Breast Cancer Res Treat.

[9] Lyman GH, Greenlee H, Bohlke K (2018). Integrative therapies during and after breast Cancer Treatment: ASCO endorsement of the SIO Clinical Practice Guideline. J Clin Oncol.

[10] Mao JJ, Tan T, Li SQ (2014). Attitudes and barriers towards participation in an acupuncture trial among breast cancer patients: a survey study. BMC Complement Altern Med.

[11] Shen X, Ding G, Wei J (2006). An infrared radiation study of the biophysical characteristics of traditional moxibustion. Complement Ther Med.

[12] Zhao L, Cheng K, Wu F (2021). Effect of laser moxibustion for knee osteoarthritis: a Multisite, double-blind Randomized Controlled Trial. J Rheumatol.

[13] Mao H, Mao JJ, Guo M (2016). Effects of infrared laser moxibustion on cancer-related fatigue: a randomized, double-blind, placebo-controlled trial. Cancer.

[14] Mao H, Mao JJ, Chen J (2019). Effects of infrared laser moxibustion on cancer-related fatigue in breast cancer survivors: study protocol for a randomized controlled trial. Med (Baltim).

[15] Wang XS, Hao XS, Wang Y (2004). Validation study of the Chinese version of the brief fatigue inventory (BFI-C). J Pain Symptom Manage.

[16] Mendoza TR, Wang XS, Cleeland CS (1999). The rapid assessment of fatigue severity in cancer patients: use of the brief fatigue inventory. Cancer.

[17] Buysse DJ, Reynolds CF, Monk TH (1989). The Pittsburgh Sleep Quality Index: a new instrument for psychiatric practice and research. Psychiatry Res.

[18] Ho RT, Fong TC (2014). Factor structure of the Chinese version of the Pittsburgh sleep quality index in breast cancer patients. Sleep Med.

[19] Li Q, Lin Y, Hu C (2016). The Chinese version of hospital anxiety and depression scale: psychometric properties in Chinese cancer patients and their family caregivers. Eur J Oncol Nurs.

[20] Lee EH (2012). Review of the psychometric evidence of the perceived stress scale. Asian Nurs Res (Korean Soc Nurs Sci).

[21] Wang XS, Mendoza TR, Gao SZ (1996). The Chinese version of the brief Pain Inventory (BPI-C): its development and use in a study of cancer pain. Pain.

[22] Cella DF, Tulsky DS, Gray G (1993). The Functional Assessment of Cancer Therapy scale: development and validation of the general measure. J Clin Oncol.

[23] Wan C, Zhang D, Yang Z (2007). Validation of the simplified Chinese version of the FACT-B for measuring quality of life for patients with breast cancer. Breast Cancer Res Treat.

[24] Mao JJ, Armstrong K, Farrar JT (2007). Acupuncture expectancy scale: development and preliminary validation in China. Explore (NY).

[25] Mao JJ, Xie SX, Bowman MA (2010). Uncovering the expectancy effect: the validation of the acupuncture expectancy scale. Altern Ther Health Med.

[26] Mao JJ, Farrar JT, Bruner D (2014). Electroacupuncture for fatigue, sleep, and psychological distress in breast cancer patients with aromatase inhibitor-related arthralgia: a randomized trial. Cancer.

[27] Han K, Kim M, Kim EJ (2021). Moxibustion for treating cancer-related fatigue: a multicenter, assessor-blinded, randomized controlled clinical trial. Cancer Med.

[28] Zhang J, Qin Z, So TH (2023). Acupuncture for chemotherapy-associated insomnia in breast cancer patients: an assessor-participant blinded, randomized, sham-controlled trial. Breast Cancer Res.

[29] Wang CC, Han EY, Jenkins M (2022). The safety and efficacy of using moxibustion and or acupuncture for cancer-related insomnia: a systematic review and meta-analysis of randomised controlled trials. Palliat Care Soc Pract.

[30] Shu Q, Wang H, Litscher D (2016). Acupuncture and moxibustion have different effects on fatigue by regulating the autonomic nervous system: a pilot controlled clinical trial. Sci Rep.

[31] Vickers AJ, Straus DJ, Fearon B (2004). Acupuncture for postchemotherapy fatigue: a phase II study. J Clin Oncol.

[32] Charalambous A, Berger AM, Matthews E (2019). Cancer-related fatigue and sleep deficiency in cancer care continuum: concepts, assessment, clusters, and management. Support Care Cancer.

[33] de Freitas LF, Hamblin MR (2016). Mechanisms of Photobiomodulation or Low-Level Light Therapy. IEEE J Sel Top Quantum Electron.

[34] Chung H, Dai T, Sharma SK (2012). The nuts and bolts of low-level laser (light) therapy. Ann Biomed Eng.

[35] Dompe C, Moncrieff L, Matys J (2020). Photobiomodulation-underlying mechanism and clinical applications. J Clin Med.

[36] O’Higgins CM, Brady B, O’Connor B (2018). The pathophysiology of cancer-related fatigue: current controversies. Support Care Cancer.

[37] Bower JE (2019). The role of neuro-immune interactions in cancer-related fatigue: Biobehavioral risk factors and mechanisms. Cancer.

[38] Li TG, Shui L, Ge DY (2019). Moxibustion reduces inflammatory response in the Hippocampus of a Chronic Exercise-Induced fatigue rat. Front Integr Neurosci.

