# Supplementary material for: Infrared laser moxibustion for cancer-related fatigue in breast cancer survivors: a randomized controlled trial
Source: Breast Cancer Res. 2024 May 21;26:80. doi: 10.1186/s13058-024-01838-1 (PMC11110353; doi:10.1186/s13058-024-01838-1)
Supplement: Supplementary file 1 — Supplementary Material 1 [file 13058_2024_1838_MOESM1_ESM.docx]

Appendix: Infrared laser moxibustion Intervention

SX10-C1 laser moxibustion devices (Shanghai Wonderful OptoElectrics Tech Co. Ltd., Shanghai, China) were performed for the ILM and SILM groups. Four laser probes were simultaneously aligned with four points (ST36 [bilateral], CV4, and CV6 acupoints) and they irradiated each acupoint 2 cm away from the skin surface for a total of 20 minutes. The ILM output power was set at 170 mW. The energy density was 64.97 J/cm2 for one treatment and the dose of per treatment spot was 203.91 J (Table 1). Each patient received this treatment twice per week for six weeks (12 session total). Patients in the SILM group adhered to the same treatment protocol as those in the ILM group. The sham laser moxibustion instrument appears to be identical to the real one. However, in the sham group, no laser was released when the instrument is turned on.

**Table 1 The Parameter of Infrared Laser Moxibustion**

| **Laser** | **Parameter** |
| --- | --- |
| **Device** | Model: SX10-C1: Wonderful Opto-Electrics Tech. Co., Ltd. (Shanghai, China) and received license from the Shanghai Municipal Food and Drug Administration, China (20162210783) |
| **Wavelength** | 10.6μm |
| **Mode** | Continuous |
| **Power output** | 170mW |
| **Energy density** | 64.97J/cm^2^ |
| **Dose** | 203.91J/ per treatment spot (2cm in diameter) |
| **Exposure time** | 20min/per treatment |
|  | (2 times/per week, total 12 sessions) |
