# Supplementary material for: Infrared laser moxibustion for cancer-related fatigue in breast cancer survivors: a randomized controlled trial
Source: Breast Cancer Res. 2024 May 21;26:80. doi: 10.1186/s13058-024-01838-1 (PMC11110353; doi:10.1186/s13058-024-01838-1)
Supplement: Supplementary file 2 — Supplementary Material 2 [file 13058_2024_1838_MOESM2_ESM.docx]

**eTable 1. The worst fatigue item and usual fatigue item in BFI across all study groups**

|  | **Mean change from baseline (95%CI)** | |  |  | | **ILM vs SILM groups** | | | | |  | **ILM vs WLC groups** | | | | |
| --- | --- | --- | --- | --- | --- | --- | --- | --- | --- | --- | --- | --- | --- | --- | --- | --- |
| **Outcome** | **ILM ( n=56)** | **SILM (n=56)** | **WLC (n=28)** | |  | | **Difference (95%CI）** | | ***^a^ P* value** | |  | | | **Difference (95%CI）** | | ***^a^ P* value** |
| **The worst fatigue item** | |  | | | | | | | | | | | | | | |
| **week 3** | -1.22 (-1.63,-0.82) | -0.80(-1.21,-0.39) | -0.30(-0.70,0.11) | | | |  | -0.44(-0.97,0.09) | | 0.102 | | |  | -0.94(-1.57,-0.31) | 0.004 | |
| **week 6** | -1.61 (-2.25,-0.97) | -1.47(-2.06,-0.87) | -0.44(-0.93,0.04) | | | |  | -0.17(-0.95,0.62) | | 0.674 | | |  | -1.19(-2.11,-0.27) | 0.012 | |
| **week 12** | -2.27(-2.29,-1.44) | -1.65(-2.22,-1.09) | -0.74(-1.20,-0.28) | | | |  | -0.63(-1.35,0.09) | | 0.086 | | |  | -1.56(-2.40,-0.71) | <.001 | |
| **week 18** | -2.57(-3.13,-2.01) | -2.00(-2.64,-1.36) | -1.07(-1.59,-0.56) | | | |  | -0.59(-1.36,0.18) | | 0.132 | | |  | -1.52(-2.44,-0.61) | 0.001 | |
| **The usual fatigue item** | |  | | | | | | | | | | | | | | |
| **week 3** | -0.86 (-1.25,-0.46) | -0.88(-1.25,-0.50) | 0.04(-0.30,0.37) | | | |  | -0.01(-0.50,0.48) | | 0.972 | | |  | -0.89(-1.47,-0.30) | 0.003 | |
| **week 6** | -1.41 (-1.89,-0.93) | -1.39(-1.88,-0.89) | -0.15(-0.65,0.35) | | | |  | -0.07(-0.70,0.56) | | 0.825 | | |  | -1.25(-1.99,-0.50) | 0.001 | |
| **week 12** | -1.92(-2.37,-1.47) | -1.63(-2.05,-1.22) | -0.41(-0.89,0.07) | | | |  | -0.32(-0.89,0.25) | | 0.270 | | |  | -1.51(-2.19,-0.84) | <.001 | |
| **week 18** | -2.16(-2.63,-1.69) | -1.73(-2.23,-1.23) | -0.67(-1.23,-0.11) | | | |  | -0.47(-1.11,0.18) | | 0.153 | | |  | -1.49(-2.26,-0.73) | <.001 | |

Abbreviations: ILM, Infrared Laser Moxibustion; SILM, Sham Infrared Laser Moxibustion; WLC, Waitlist Control; BFI, Brief Fatigue Inventory.

^a^ *P* values were calculated using a mixed-effects model.

**eTable 2. Each domain scores in FACT-B across all study groups**

|  | **Mean change from baseline (95%CI)** | | | |  |  | | **ILM vs SILM groups** | | | | | | |  | | | **ILM vs WLC groups** | | | | | | | |  |  |
| --- | --- | --- | --- | --- | --- | --- | --- | --- | --- | --- | --- | --- | --- | --- | --- | --- | --- | --- | --- | --- | --- | --- | --- | --- | --- | --- | --- |
| **Outcome** | **ILM ( n=56)** | | **SILM (n=56)** | | **WLC (n=28)** | | | |  | **Difference (95%CI）** | | | ***^a^ P* value** | | | |  | | | | **Difference (95%CI）** | | | ***^a^ P* value** | | | |
| **PWB** | | |  | | | | | | | | | | | | | | | | | | | | | | |  |  |
| **week 3** | 1.16 (0.13,2.20) | | 0.57(-0.10,1.25) | | 0.59(0.09,1.10) | | | |  | 0.46(-0.60,1.53) | | 0.394 | | | | | | |  | | | 0.47(-0.80,1.74) | 0.465 | | | |  |
| **week 6** | 2.00 (0.74,3.26) | | 1.14(0.42,1.87) | | 0.37(-0.29,1.03) | | | |  | 0.69(-0.56,1.95) | | 0.276 | | | | | | |  | | | 1.51(0.01,3.00) | 0.048 | | | |  |
| **week 12** | 2.53(1.27,3.79) | | 1.55(0.79,2.31) | | 0.81(-0.19,1.82) | | | |  | 0.81(-0.51,2.12) | | 0.226 | | | | | | |  | | | 1.60(0.04,3.17) | 0.045 | | | |  |
| **week 18** | 2.35(1.10,3.60) | | 1.39(0.50,2.28) | | 1.07(-0.09,2.24) | | | |  | 0.77(-0.62,2.17) | | 0.273 | | | | | | |  | | | 1.14(-0.52,2.18) | 0.177 | | | |  |
| **SWB** | | |  | |  | | | |  |  | |  | | | | | | |  | | |  |  | | | |  |
| **week 3** | 0.49 (-0.34,1.32) | | 0.71(-0.04,1.47) | | 0.07(-0.19,0.34) | | | |  | -0.21(-1.19,0.77) | | 0.676 | | | | | | |  | | | 0.45(-0.71,1.61) | 0.447 | | | |  |
| **week 6** | 0.63 (-0.19,1.45) | | 0.00(-1.07,1.07) | | 0.26(-0.24,0.76) | | | |  | 0.65(-0.53,1.84) | | 0.278 | | | | | | |  | | | 0.39(-1.01,1.78) | 0.582 | | | |  |
| **week 12** | 0.53(-0.31,1.37) | | 0.76(-0.08,1.59) | | 0.37(-0.18,0.92) | | | |  | -0.20(-1.25,0.85) | | 0.708 | | | | | | |  | | | 0.22(-1.02,1.45) | 0.730 | | | |  |
| **week 18** | 0.90(-0.07,1.87) | | 0.33(-0.48,1.14) | | -0.04(-0.68,0.61) | | | |  | 0.60(-0.54,1.73) | | 0.299 | | | | | | |  | | | 0.97(-0.37,2.31) | 0.154 | | | |  |
| **EWB** | | |  | |  | | | |  |  | |  | | | | | | |  | | |  |  | | | |  |
| **week 3** | 0.51(-0.43,1.45) | | 0.16(-0.42,0.74) | | -0.22(-1.03,0.59) | | | |  | 0.35(-0.66,1.37) | | 0.492 | | | | | | |  | | | 0.73(-0.48,1.94) | 0.237 | | | |  |
| **week 6** | 1.08 (-0.02,2.18) | | 0.51(-0.36,1.38) | | 0.22(-0.51,0.95) | | | |  | 0.58(-0.63,1.80) | | 0.341 | | | | | | |  | | | 0.85(-0.59,2.29) | 0.244 | | | |  |
| **week 12** | 1.27(0.24,2.29) | | 1.20(0.31,2.10) | | 0.30(-0.35,0.94) | | | |  | 0.07(-1.09,1.24) | | 0.900 | | | | | | |  | | | 0.97(-2.06,-0.76) | 0.173 | | | |  |
| **week 18** | 1.49(0.49,2.49) | | 1.18(-0.01,2.37) | | 0.26(-0.43,0.95) | | | |  | 0.32(-1.02,1.66) | | 0.638 | | | | | | |  | | | 1.18(-0.43,2.78) | 0.150 | | | |  |
| **FWB** | | |  | |  | | | |  |  | |  | | | | | | |  | | |  |  | | | |  |
| **week 3** | 1.08 (-0.21,2.37) | | 0.82(0.00,1.63) | | -0.44(-1.02,0.13) | | | |  | 0.17(-1.17,1.51) | | 0.805 | | | | | | |  | | | 1.37(-0.22,2.96) | 0.091 | | | |  |
| **week 6** | 1.57 (0.19,2.96) | | 0.63(-0.83,2.10) | | -0.30(-1.23,0.63) | | | |  | 0.81(-0.97,2.59) | | 0.370 | | | | | | |  | | | 1.68(-0.42,3.78) | 0.116 | | | |  |
| **week 12** | 1.51(0.14,2.88) | | 0.98(-0.09,2.05) | | -0.11(-1.27,1.05) | | | |  | 0.41(-1.18,1.99) | | 0.612 | | | | | | |  | | | 1.45(-0.42,3.32) | 0.128 | | | |  |
| **week 18** | 1.65(0.33,2.97) | | 0.45(-0.92,1.82) | | -0.07(-1.22,1.08) | | | |  | 1.07(-0.64,2.79) | | 0.219 | | | | | | |  | | | 1.53(-0.51,3.56) | 0.140 | | | |  |
| **BCS** | | |  | |  | | | |  |  | |  | | | | | | |  | | |  |  | | | |  |
| **week 3** | 0.63 (-0.45,1.71) | | 0.43(-0.54,1.40) | | 0.04(-0.49,0.57) | | | |  | 0.18(-1.08,1.45) | | 0.774 | | | | | | |  | | | 0.55(-0.95,2.05) | 0.472 | | | |  |
| **week 6** | 1.45 (0.20,2.69) | | 0.51(0.42,1.44) | | 0.67(-0.28,1.61) | | | |  | 0.91(-0.47,2.29) | | 0.193 | | | | | | |  | | | 0.73(-0.91,2.37) | 0.380 | | | |  |
| **week 12** | | 1.43(0.29,2.57) | 0.76(-0.24,1.75) | 0.85(-0.24,1.95) | | |  | | 0.65(-0.72,2.01) | | | | | 0.350 | |  | | | | 0.53(-1.09,2.15) | | | | | 0.520 |  |  |
| **week 18** | | 1.47(0.35,2.59) | 1.04(-0.06,2.14) | 1.22(0.21,2.24) | | |  | | 0.40(-1.00,1.80) | | 0.572 | | | | |  | | | | 0.18(-1.49,1.84) | | | | | 0.835 |  |  |

Abbreviations: ILM, Infrared Laser Moxibustion; SILM, Sham Infrared Laser Moxibustion; WLC, Waitlist Control; FACT-B: Functional Assessment of Cancer Therapy-Breast; PWB: physical well-being subscale of FACT-B; SWB: social well-being subscale of FACT-B; EWB: emotional well-being subscale of FACT-B; FWB: functional well-being subscale of FACT-B; BCS: breast cancer subscale of FACT-B.

^a^ *P* values were calculated using a mixed-effects model.

**eTable 3. Chang of scores in HADS, PSS-10 and BFI across all study groups**

|  | **Mean change from baseline (95%CI)** | |  |  | | **ILM vs SILM groups** | | | | |  | | **ILM vs WLC groups** | | | |
| --- | --- | --- | --- | --- | --- | --- | --- | --- | --- | --- | --- | --- | --- | --- | --- | --- |
| **Outcome** | **ILM ( n=56)** | **SILM (n=56)** | **WLC (n=28)** | |  | | **Difference (95%CI）** | | | ***^a^ P* value** |  | | **Difference (95%CI）** | | | ***^a^ P* value** |
| **HADS-A** | |  | | | | | | | | | | | | | | |
| **week 3** | -0.63(-1.42,0.15) | -0.39(-0.94,0.17) | -0.56(-1.18,0.07) | |  | | | -0.28(-1.15,0.59) | 0.524 | | |  | | -0.06(-1.10,0.97) | 0.902 | |
| **week 6** | -1.57(-2.47,-0.67) | -1.06(-1.69,-0.44) | -0.74(-1.49,0.01) | |  | | | -0.57(-1.55,0.41) | 0.253 | | |  | | -0.79(-1.96,0.37) | 0.181 | |
| **week 12** | -1.92(-2.89,-0.95) | -1.65(-2.42,-0.89) | -0.89(-1.83,0.06) | |  | | | -0.34(-1.45,0.77) | 0.549 | | |  | | -0.95(-2.28,0.38) | 0.158 | |
| **week 18** | -2.43(-3.49,-1.36) | -1.20(-2.16,-0.25) | -1.19(-2.23,-0.14) | |  | | | -1.31(-2.60,0.01) | 0.048 | | |  | | -1.10(-2.65,0.44) | 0.160 | |
| **HADS-D** | |  |  | |  | | |  |  | | |  | |  |  | |
| **week 3** | -0.35(-1.01,0.32) | -0.10(-0.56,0.36) | -0.11(-0.48,0.26) | |  | | | -0.25(-0.97,0.47) | 0.488 | | |  | | -0.24(-1.09,0.61) | 0.581 | |
| **week 6** | -0.88(-1.68,-0.07) | -0.49(-1.14,0.16) | -0.44(-1.13,0.24) | |  | | | -0.40(-1.34,0.53) | 0.395 | | |  | | -0.45(-1.56,0.66) | 0.426 | |
| **week 12** | -1.35(-2.21,-0.48) | -0.84(-1.34,-0.34) | -0.33(-0.91,0.25) | |  | | | -0.53(-1.42,0.53) | 0.245 | | |  | | -0.95(-2.01,0.10) | 0.077 | |
| **week 18** | -1.55(-2.51,-0.59) | -0.55(-1.52,0.42) | -0.59(-1.12,-0.06) | |  | | | -1.02(-2.22,0.18) | 0.096 | | |  | | -0.88(-2.31,0.55) | 0.226 | |
| **PSS-10** | |  |  | |  | | |  |  | | |  | |  |  | |
| **week 3** | -1.61(-3.09,-0.13) | -1.27(-2.53,0.00) | -1.04(-2.45,0.38) | |  | | | -0.33(-2.12,1.47) | 0.720 | | |  | | -0.61(-2.74,1.53) | 0.575 | |
| **week 6** | -2.69(-4.49,-0.90) | -1.53(-3.08,0.02) | -0.96(-2.34,0.42) | |  | | | -1.13(-3.23,0.98) | 0.291 | | |  | | -1.79(-4.30,0.71) | 0.159 | |
| **week 12** | -2.92(-4.80,-1.04) | -3.31(-5.06,-1.55) | -1.30(-3.15,0.56) | |  | | | 0.43(-1.89,2.75) | 0.713 | | |  | | -1.70(-4.47,1.06) | 0.225 | |
| **week 18** | -3.55(-5.70,-1.40) | -2.76(-4.64,-0.87) | -1.07(-2.81,0.66) | |  | | | -0.75(-3.28,1.78) | 0.559 | | |  | | -2.51(-5.54,0.52) | 0.104 | |
| **BPI** | |  |  | |  | | |  |  | | |  | |  |  | |
| **week 3** | -0.10(-0.33,0.13) | -0.22(-0.61,-0.17) | -0.23(-0.51,0.05) | |  | | | 0.15(-0.26,0.56) | 0.469 | | |  | | 0.13(-0.36,0.62) | 0.596 | |
| **week 6** | -0.48(-0.94,0.03) | -0.32(-0.74,0.11) | -0.20(-0.68,0.28) | |  | | | -0.09(-0.66,0.47) | 0.745 | | |  | | -0.26(-0.94,0.41) | 0.443 | |
| **week 12** | -0.42(-0.73,-0.10) | -0.61(-0.99,-0.23) | -0.32(-0.91,0.27) | |  | | | 0.25(-0.24,0.75) | 0.314 | | |  | | -0.08(-0.67,0.51) | 0.779 | |
| **week 18** | -0.53(-0.89,-0.17) | -0.62(-1.06,-0.18) | -0.40(-0.79,0.00) | |  | | | 0.15(-0.36,0.67) | 0.560 | | |  | | -0.13(-0.74,0.49) | 0.686 | |
| **AES** |  |  |  | |  | | |  |  | | |  | |  |  | |
| **week 3** | 0.18(-0.94,1.31) | - 0.88(-1.95,0.20) | / | |  | | | 1.00(-0.50,2.50) | 0.188 | | |  | | / | / | |
| **week 6** | -0.82(-2.17,0.54) | -0.58(-1.77,0.60) | / | |  | | | -0.26(-2.01,1.48) | 0.765 | | |  | | / | / | |

Abbreviations: ILM, Infrared Laser Moxibustion; SILM, Sham Infrared Laser Moxibustion; WLC, Waitlist Control;HADS, Hospital Anxiety and Depression Scale; PSS-10, 10-item Perceived Stress Scale; BPI, Brief Pain Inventory; AES, Acupuncture expectancy Scale.

^a^ *P* values were calculated using a mixed-effects model.
